# A Rare Presentation of *Plasmodium vivax* Malaria: Pancytopenia due to Hemophagocytic Syndrome in a Pregnant Woman

**DOI:** 10.1155/carm/1035584

**Published:** 2025-07-02

**Authors:** Girma Deshimo Lema, Asrat Berihun Dagnaw, Enguday Demeke Gebeyaw

**Affiliations:** ^1^School of Medicine, Department of Internal Medicine, Asrat Woldeyes Health Science Campus, Debre Berhan University, Debre Berhan, Ethiopia; ^2^School of Public Health, Department of Epidemiology and Biostatistics, Asrat Woldeyes Health Science Campus, Debre Berhan University, Debre Berhan, Ethiopia

**Keywords:** hemophagocytic syndrome, malaria, pancytopenia, *Plasmodium vivax*, pregnancy

## Abstract

**Background:** Malaria poses a significant public health challenge, especially in pregnant women, due to potential complications for both mother and fetus. The occurrence of pancytopenia as an initial manifestation of acute *Plasmodium vivax* malaria is extremely rare, with most cases reported in *Plasmodium falciparum (P*. *falciparum)* malaria infections. To the best of our knowledge, there are no documented cases of pancytopenia due to hemophagocytic syndrome (HPS) associated to *P*. *vivax* malaria in pregnant women.

**Case Presentation:** A 22-year-old gravida one woman from Debre Berhan, Ethiopia, who was nine weeks pregnant, presented with a high-grade fever, headache, nausea, vomiting, joint pain, and fatigue lasting for 1 week. She had recently traveled to a malaria-endemic region. Upon examination and investigation, she was found to be hypotensive and febrile, with pale conjunctiva, hepatosplenomegaly, and pancytopenia, along with elevated levels of triglycerides and serum ferritin. Blood smear analysis showed the trophozoite and gametocyte stages of *P*. *vivax*. She was diagnosed with HPS based on clinical criteria. The patient achieved full recovery following antimalarial and supportive treatment.

**Conclusion:** This case highlights a rare but serious presentation of *P. vivax* malaria in a pregnant woman, characterized by HPS and resultant pancytopenia. Timely diagnosis and effective treatment are crucial for achieving good outcomes for both the mother and the fetus, highlighting the importance of increased awareness in clinical practice.

## 1. Introduction

Malaria is a vector-borne parasitic disease caused by protozoan parasites from the genus Plasmodium, predominantly found in tropical and subtropical regions around the world [1]. Malaria is one of the most critical public health issues globally. It is a significant cause of illness and death in many developing countries, particularly impacting young children and pregnant women [2]. Worldwide, the highest mortality rates from malaria are associated with *P*. *falciparum* infection. However, non-falciparum malaria can also result in considerable morbidity and mortality, including clinical deterioration after initiation of treatment [3–5]. *Plasmodium vivax* is the second most prevalent cause of human malaria, following *P. falciparum*. In 2022, there were approximately 6.9 million reported cases of *P. vivax* in the world [2]. About 82% of the global burden of *P*. *vivax* comes from four high-burden countries: India, Pakistan, Ethiopia, and Sudan [6]. *P. vivax* poses challenges for control measures due to its dormant liver stages, known as hypnozoites, which can lead to relapses of the infection even months after the initial episode [7].

Around 75% of Ethiopia's land is at risk for malaria, affecting 69% of the population in those areas [8]. Within the country, *P*. *falciparum* predominates as the main parasite, following *P*. *vivax* [9]. Ethiopia's ecological diversity contributes to a wide range of malaria transmission dynamics, varying from low-risk areas to regions with high transmission rates [10]. The country is actively implementing a malaria elimination program [11].

The clinical manifestations of *P. vivax* infection are typically less severe than those of *P*. *falciparum*; however, there are occasional cases that can result in serious complications [12–15]. Pancytopenia is an exceedingly rare complication of acute *P*. *vivax* malaria, with several proposed mechanisms including macroangiopathic hemolytic anemia, hemophagocytic syndrome (HPS), and direct suppression of bone marrow function [16–19]. HPS is a rare clinicopathological condition marked by excessive immune system activation, leading to the inappropriate phagocytosis of erythroid precursors, granulocytes, and platelets, as well as multiorgan dysfunction. HPS is classified into inherited and acquired forms. Acquired HPS primarily arises as a secondary condition due to various infections, including viral, bacterial, fungal, and parasitic infections. Additionally, autoimmune disorders and hematological malignancies can also lead to HPS [20–23].

In pregnant women, malaria can have devastating effects, including increased risks of maternal morbidity, fetal loss, and low birth weight [24–28]. The presence of HPS complicates this picture, as it can lead to significant hematological abnormalities and multiorgan dysfunction. Recognizing the rare presentation of *P*. *vivax* malaria in pregnant women as a cause of pancytopenia due to HPS is essential for early diagnosis and management, ultimately enhancing maternal and fetal outcomes. This report presents a case of pancytopenia due to hemphagocytic syndrome associated with *P*. *vivax* malaria in a pregnant woman, which, to the best of our knowledge, has not been previously documented.

## 2. Case Presentation

A 22-year-old pregnant woman from Debre Berhan, located in northeast Ethiopia, presented to Debre Berhan University Hakim Gizaw Hospital with a one-week history of high grade fever, headache, chills, nausea, vomiting, arthralgia, and fatigue. She was gravida one and 9 weeks pregnant. Although she resides in a nonendemic area for malaria, she had traveled to the southern part of Ethiopia, a malaria-endemic region, where she stayed for 2 weeks and returned 10 days prior to her presentation. She reported no history of similar medical condition in the past. She had no cough, shortness of breath, abdominal pain, diarrhea, or change in mentation. Her family history was unremarkable.

On physical examination, her blood pressure was 85/51 mmHg, pulse rate was 104 beats per minute, respiratory rate was 24 breaths per minute, temperature was 38.8°C, and oxygen saturation was 94% on room air. She had dry buccal mucosa and pale conjunctiva. The spleen was palpable 1 cm below the left lower costal margin, while all other examinations were unremarkable.

**Figure 1:**
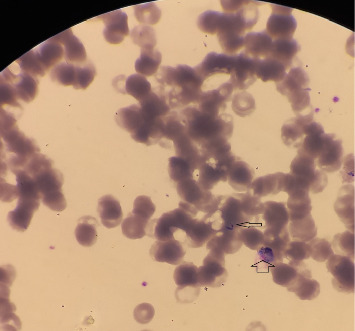
Peripheral blood film displaying the trophozoite (thin arrow) and gametocyte (wide arrow) stages of *P*. *vivax*.

Laboratory examination revealed pancytopenia on the complete blood count (CBC), with white blood cell count at 2.3 × 10^3^/mm^3^, hemoglobin at 8.1 g/dL, mean corpuscular volume (MCV) at 88.5 fL, and platelet count at 43 × 10^3^/mm^3^. The liver function test showed a mild elevation, while random blood sugar, renal function tests, serum electrolytes, and urinalysis were all within normal ranges. Serologic tests for hepatitis and human immunodeficiency virus (HIV) were negative. Additional investigations and follow-up CBC results are summarized in Table 1. A peripheral blood film revealed the presence of trophozoites and gametocytes of *P. vivax* (Figure 1). The species-specific rapid diagnostic test (RDT) was positive for *P*. *vivax* and negative for *P*. *falciparum*. Abdominopelvic ultrasonography revealed hepatomegaly (16.7 cm), splenomegaly (14.5 cm), and a viable intrauterine pregnancy with a gestational age of 9 weeks and 5 days.

She was treated with intravenous artesunate because her vomiting prevented her from tolerating oral antimalarials, and she had signs of severity. Intravenous fluids were administered for resuscitation, along with other supportive care, including antipyretic (paracetamol) and glucose monitoring. The patient responded well to the treatment, with her vital signs stabilizing and her fever subsiding within 48 h. After 4 days, she was discharged on chloroquine with a follow-up appointment scheduled for 1 week later. During her follow-up visit, her hematologic parameters had normalized, as shown in Table 1. The patient was monitored closely throughout the pregnancy, the fetus remained viable with no signs of distress, and no congenital anomalies were detected during routine ultrasound evaluations. Due to the pregnancy, primaquine was not administered during hospitalization, in accordance with the current guidelines; however, the patient was counseled to receive primaquine after delivery for radical cure.

## 3. Discussion

Malaria is a significant global health issue in many countries, particularly in tropical and subtropical regions, including sub-Saharan Africa, Asia, and Latin America [2]. Regardless of various prevention and control strategies, malaria continues to be a significant public health problem in Ethiopia [29].

*P. vivax* malaria is often viewed as less severe compared to other malaria species, yet this case demonstrates its potential for serious complications, particularly in pregnant women. The occurrence of pancytopenia due to HPS is particularly rare, even in cases of *P*. *falciparum* malaria, highlighting the complexities of malaria in this vulnerable population.

### 3.1. Mechanism of Pancytopenia

The potential mechanisms of pancytopenia in the case of malaria likely include immune activation, bone marrow suppression, splenic sequestration, and direct hemolysis. However, the most probable cause in our case is HPS.

HPS is a life-threatening hematologic condition characterized by the uncontrolled activation of immune cells. In this state, activated macrophages can phagocytize not only infected erythrocytes but also healthy blood cells, leading to pancytopenia [21, 23]. HPS often results in multiorgan dysfunction and presents with a wide range of nonspecific symptoms. Cardinal manifestations include fever, hepatosplenomegaly, lymphadenopathy, neurologic symptoms, and/or rash. Common laboratory findings in patients with HPS include high serum ferritin levels, pancytopenia, elevated liver function tests, increased D-dimer, hypertriglyceridemia, and hypofibrinogenemia [30–32]. In our case, the patient had fever, hepatosplenomegaly, pancytopenia, and elevated serum triglyceride and ferritin levels, consistent with HPS according to the revised diagnostic criteria guideline of the HLH-2004 protocol [33] (Table 2).

In nonpregnant patients, pancytopenia associated with HPS in *P*. *vivax* malaria has been reported in the literature [18, 19, 22, 35], as well as a case of HPS leading to bicytopenia [36]. Additionally, pancytopenia has been noted in *P*. *vivax*, although the mechanism has not been mentioned [16, 37].

In a case series and literature review on HPS in pregnancy by Jessica Parrott et al., it was found that HIV and *P*. *falciparum* were present in one of the 26 cases reported [38]. However, the authors concluded that HIV infection, rather than malaria, was the cause of HPS in this patient, as she did not respond to antimalarial treatment [39].

Although malaria can cause hemolysis of infected red blood cells, the degree of hemolysis alone typically results in anemia rather than the full spectrum of pancytopenia seen here. In this case, the prominent presence of leukopenia and thrombocytopenia, alongside anemia, suggests a more systemic process rather than isolated hemolysis. Additionally, the clinical presentation of HPS, with its associated immune activation, indicates that a broader mechanism is at play. In cases of splenic sequestration, the spleen often enlarges and retains blood cells, which would typically lead to more pronounced anemia rather than a decrease in all three cell lines. Yamakawa et al. reported a case of pancytopenia in a patient with *P. vivax* that was attributed to bone marrow hypoplasia [40].

In summary, although hemolysis, splenic sequestration, and bone marrow suppression may contribute to pancytopenia in malaria, the clinical presentation and laboratory findings in this case are consistent with HPS.

### 3.2. Challenges in Pregnant Woman

Pregnant women are particularly vulnerable to severe malaria, which presents significant health risks for both the mother and fetus [26, 41, 42]. *P*. *vivax* malaria can result in anemia, hypoglycemia, and severe illness, increasing the risk of maternal mortality. The infection can lead to adverse perinatal outcomes such as low birth weight, premature delivery, and increased neonatal mortality. In some cases, it can cause placental malaria, which affects fetal nutrition and growth [4, 16, 25, 27, 43, 44]. The presence of HPS in pregnant patients with malaria significantly worsens the prognosis, making timely diagnosis and management crucial. In our case, the patient was in her first trimester of pregnancy, and there were no immediate adverse outcomes observed. Factors such as the timing of the infection, the patient's immune response, and the management of the malaria episode can influence outcomes during pregnancy. Continued monitoring and care are essential to ensure maternal and fetal health throughout the course of the pregnancy as early infections can lead to delayed effects on maternal and fetal health.

### 3.3. Management

Effective management necessitates prompt treatment of the malaria infection with appropriate antimalarials, which can help reduce the parasitic load and, consequently, the hyperinflammatory response. Our case responded well to antimalarial and supportive care. Previous reports have proven that malaria-associated HPS has excellent response with such approach without any need for immunosuppressant and steroids [16–18, 37]. HPS related with other causes have several options of treatment [45].

## 4. Conclusion

This case highlights the critical need to recognize atypical presentations of *P*. *vivax* malaria, particularly in vulnerable populations such as pregnant women. Although *P*. *vivax* is often considered less severe than other malaria species, this case demonstrates its potential to cause serious complications, including pancytopenia due to HPS. The complexities of malaria during pregnancy necessitate a high level of clinical suspicion for severe manifestations, especially given the significant health risks for both mother and fetus. Prompt diagnosis and effective management, including the timely use of appropriate antimalarials and supportive care, are essential to mitigate complications and improve outcomes.

## Figures and Tables

**Table 1:** Summary of laboratory results.

Laboratory tests	At admission	After 36 h	After 3 days	After 12 days
CBC				
WBC	2.3 × 10^3^/mm^3^	2.5 × 10^3^/mm^3^	3 × 10^3^/mm^3^	6.8 × 10^3^/mm^3^
Hgb	8.1 g/dL	8.3 g/dL	9 g/dL	11.4 g/dL
Plt	37 × 10^3^/mm^3^	43 × 10^3^/mm^3^	104 × 10^3^/mm^3^	190 × 10^3^/mm^3^
GOT	114 u/L	—	92 u/L	34 u/L
GPT	68 u/L	—	58 u/L	25 u/L
Total bilirubin	2.2 mg/dL	—	—	0.94 mg/dL
Direct bilirubin	1.8 mg/dL	—	—	0.25 mg/dL
Triglyceride	220 mg/dL	—	—	160 mg/dL
LDH	614 u/L	—	—	236 u/L
CRP	68 mg/dL	—	—	< 5 mg/dL
Ferritin	560 ng/mL	—	—	292 ng/dL
Fibrinogen	NA			

*Note:* Hgb, hemoglobin; Plt, platelet; GOT, aspartate aminotransferase; LDH, lactate dehydrogenase; GPT, alanine aminotransferase.

Abbreviations: CRP, C reactive protein; NA, not available; WBC, white blood count.

**Table 2:** Diagnostic criteria for hemophagocytic syndrome/hemophagocytic lymphohistiocytosis [34]^∗^.

Genetic defect consistent with HPS
Or
The presence of at least five of the eight of the following features
1. Fever ≥ 38.5°C
2. Splenomegaly
3. Cytopenia = > 2 cell lines
• Hemoglobin < 9 g/dL
• Platelets < 100 × 10^3^/mm^3^
• Neutrophils < 1 × 10^3^/mm^3^
4. Hypertriglyceridemia and/or hypofibrinogenemia
5. Hemophagocytosis in bone marrow, spleen, lymph node, or liver
6. Low or absent NK cell activity
7. Ferritin > 500 ng/mL
8. Elevated soluble CD25 level

^∗^Supportive evidence is lymph node enlargement, rash, edema, central nervous system symptoms, hypoproteinemia, hyponatriemia, elevation of liver transaminases, lactate dehydrogenase, and cell count and protein of cerebrospinal fluid.

## Data Availability

The data that support this case report are within the manuscript.

## Nomenclature

CBC: Complete blood count
HPS: Hemophagocytic syndrome
